# Mental Nurses in Military Hospitals

**Published:** 1916-10-07

**Authors:** 


*To the Editor of* The Hospital.

### Mental Nurses in Military Hospitals.

*To the Editor of* The Hospital.

Sir,—In your notes on this subject, appearing in your issue of September 30, I see that I am stated to have conceded all that was asserted in previous comments. I really have conceded nothing. With regard to the main question—that of the scope of the Medico-Psychological Association—neither I nor "Justice" have raised any question in connection with it that can be arguable. A mere recognition of demonstrable fact must not be regarded as concession. I make a point of this, since I still think that the object of "Justice's" letter has been misconceived.

I assuredly will never concede the accuracy of the other subsidiary contention as to the social position of one or other class of nurse. I deny that any person or persons can form, from their own knowledge, an accurate opinion on a subject which can only be decided by statistics. My own belief, after a very considerable experience, is that the difference in matter of origin between general and mental hospital nurses is very much the difference between Tweedledum and Tweedledee. It is true that some women of the upper classes enter both institutions to learn nursing, paying for their tuition in general hospitals. But these, of course, do not affect the point at issue.

It is reassuring to read that the choice between hospital and asylum is chiefly influenced by money, and not, as one might have feared, by a sense of superiority of calling or person. The contrast which you draw between the advantages and disadvantages of the two forms of nursing is on the whole excellent, and it should be of value in helping to fill the ranks of the asylum staff. But I cannot agree to the view that the work in the asylum is less arduous or the discipline less strict than is the case with the general hospital. Certainly, as to the latter, in addition to the penalty of dismissal for breach, common to both, the penalties incurred under the lunacy law ensures the utmost care and the strictest discipline throughout the asylum.

I beg to suggest that all allegations of personal or social differences are to be strenuously deprecated at the present moment, when the whole nursing world is apparently about to be remodelled. One hears and reads of contention everywhere, contention in the matters of registration, of a College of Nursing, of uniforms, of relative positions, and so forth, giving the impression that each is trying to push the neighbour off the raft. It is possible that eventually the nursing profession may benefit by thus going through fire needlessly, but it certainly must suffer much in the process. It is easy to forget in the study of self that the profession of nursing has been evolved in the interest of the sick and not of the nurse.

In conclusion, I beg to acknowledge the compliment of being held to have been very temperate in my former communication. I was not, however, aware that any other spirit was to be regarded as possible.—Yours faithfully, Your Correspondent of September 16.

